# Tendencies of eating disordered behaviours in male content creators: a social media analysis

**DOI:** 10.1186/s40337-025-01395-8

**Published:** 2025-09-09

**Authors:** Alina Schmitt, Marius Frenser, Tobias Fischer

**Affiliations:** Center for Nutrition and Therapy (NuT), University of Applied Sciences Muenster, Corrensstraße 25, 48149 Muenster, Germany

**Keywords:** Eating disorders, Anorexia nervosa, Muscle dysmorphia, Social media analysis, Instagram, TikTok, Content creator

## Abstract

Eating disorders are primarily associated with women and an obsession with thinness. Recent research and social media content show that men are also concerned about their body image, striving for a muscular and athletic physique. To investigate eating disorder tendencies among male content creators with a mesomorphic body type (*N* = 26), a social media analysis was conducted on Instagram and TikTok over four weeks. Published content and profiles were screened using ICD-11 diagnostic criteria for anorexia nervosa, which were then adapted to take account of gender differences. A total of 2,985 posts were recorded, of which 69% were classified as relevant to diet, exercise and body content. Six content creators (23%) met the ICD-11 diagnostic criteria for anorexia nervosa, while gender-specific adaptations identified 15 individuals (58%) with tendencies toward an eating disorder. The profile analysis shows that gender-specific adaptation of the diagnostic criteria with regard to the pursuit of muscularity can facilitate the additional recognition of eating disorder-related behaviours in males. Male children and adolescents should be educated at an early stage to avoid critical behaviour patterns and the internalisation of utopian beauty ideals.

**Supplementary Information:**

The online version contains supplementary material available at 10.1186/s40337-025-01395-8.

## Abstract (plain language summary)

Eating disorders are primarily associated with women and the goal of achieving a slim body. However, men can also feel under pressure to maintain an attractive physique, particularly to appear muscular and athletic. Social media plays a major role in shaping these expectations. This analysis of social media examined 26 male content creators on Instagram and TikTok who were already muscular and athletic. Over four weeks, almost 3,000 of their posts were analysed for themes relating to diet, exercise and body image. Standard medical criteria for anorexia nervosa were applied and adapted to address male-specific concerns, in order to evaluate tendencies towards eating disorders. The results showed that six men (23%) met the official criteria for anorexia nervosa. Using the adapted criteria, it was found that 15 men (58%) displayed tendencies of disordered eating or exercise behaviour. These results suggest that current diagnostic tools may overlook important male-specific symptoms. Providing early education to boys and young men could help prevent them from adopting unrealistic body ideals and engaging in risky behaviours.

**Supplementary Information:**

The online version contains supplementary material available at 10.1186/s40337-025-01395-8.

## Introduction

The average time spent on social media by individuals under the age of 30 is reported to be 69 min per day [1]. According to the results of the Pew Research Centre’s Global Attitudes Spring 2023 Survey, social media use is almost evenly distributed between both genders worldwide, with the proportion of female non-users (56.9%) higher than that of male non-users (43.1%) [2]. In addition, between 2015 and 2024, U.S. teenagers aged 13 to 17 showed increasing user numbers on Instagram (2024: 61%), TikTok (2024: 63%) and Snapchat (55%), with TikTok having the highest user rate. After YouTube (72%), TikTok is visited daily by 57% of young people, followed by Instagram (50%) [3]. The platform’s content encompasses a diverse range of subjects, with a notable prevalence of nutrition and health-related posts [4, 5]. The prevalence of such content is evidenced by the hashtag #whatieatinaday (what-I-eat-in-a-day), which has amassed over a million posts [6]. In this context, content creators share a variety of content, including nutritional habits and sports-related content, using hashtags such as #musclegain or #fitness [7, 8]. In doing so, content creators often deliberately present themselves in revealing clothing in order to generate more likes, interactions and thus an increasing reach [9]. A notable trend among male content creators is the pursuit of a defined body type [10, 11], while the ideal for female content creators remains a slim figure [12, 13]. The recommendations for achieving these ideals are often accepted uncritically by consumers [14, 15]. Various social media posts, including images of scantily clad bodies, calorie information on food or various fitness-related posts, can generally be categorised as non-eating disorder-specific, as they are considered unproblematic for mentally healthy individuals [16]. For individuals already affected by eating disorders, these materials can pose a comparable risk to eating disorder-specific content, which encompasses, for instance, content that glamorizes the illness or recovery accounts [17, 18]. The dissemination of physical ideals and unrealistic beauty standards by social media is well-documented. There is a demonstrable link between exposure to such content and increased body dissatisfaction among consumers, with the resultant comparison processes being a key factor [16, 19]. A video-based cross-sectional analysis showed that content on TikTok is highly influenced by diet culture and often normalises practices such as body checking [20]. In addition, weight-normative messages predominate on the platform, which could potentially promote body dissatisfaction and eating disorders [21]. Given the proliferation of social media content pertaining to the body, there has been a concomitant rise in research interest in the subjects of body dissatisfaction, disturbed body image perception and their impact on eating behaviour, with the female gender being the primary focus of these studies [22, 23]. Nevertheless, it is obvious that social media also demonstrates that men allocate a significant amount of attention to their body image. A systematic review suggests that male adolescents with an Instagram account exhibit disparities in body image and eating behaviour compared to their peers without an account [24]. In this context, restrictive lifestyles are often promoted as a means of achieving the desired body type, characterised by broad shoulders, a slim waist, and defined muscles [25, 26]. Such a restrictive lifestyle typically involves strict dietary control and obsessive exercise [25], behaviours that resemble those commonly observed in anorexia nervosa, such as including restrictive eating, excessive preoccupation with food, and pronounced concerns about body weight and shape [26, 27]. In recent years, the pursuit of muscle has been further researched and body dysmorphic disorder, also known as muscle dysmorphia [28] or reverse anorexia [29], has been identified [30]. This disorder is classified as an obsessive-compulsive disorder and is characterised by a persistent preoccupation with perceived flaws in one’s appearance, such as a fear of not being muscular enough [28]. In view of the restrictive lifestyle described in the literature [25] and the frequent occurrence of body dysmorphia [28], both of which are also characteristic of anorexia nervosa [27], the following will refer to this eating disorder. Although case reports of male patients with anorexia nervosa have been documented since 1689, the male gender was largely neglected in research on eating disorders until the 20th century. Those affected were predominantly characterised as women between the ages of 16 and 23 [31, 32]. To this day, the disorder is stigmatised in society as a typical female disorder [33], although it can be assumed that there are a large number of unreported cases of male sufferers [34]. In addition, factors such as sexual orientation appear to play a relevant role. Sexual minorities such as homosexual men in particular seem to be at increased risk of eating disorders [35, 36]. The gendered stigma can discourage men from seeking help, thereby creating additional barriers to accessing appropriate diagnosis and treatment. As a result, symptoms may remain untreated for longer periods, increasing the risk of chronicity and more severe health consequences [33]. Anorexia nervosa is defined as a mental illness in the form of an eating disorder that involves low body weight and the motivation to maintain that weight. The diagnostic criteria of the International Classification of Diseases (ICD), 11th version, are utilised as a foundation for identifying and diagnosing anorexia nervosa [27]. The behaviours listed in ICD-11 correspond to the classic manifestation in female sufferers, which is predominantly characterised by rapid weight loss and a low body weight [34]. These criteria frequently exclude affected men who pursue the ideal of an athletic, slim body, and as a result, they are not generally covered by the current diagnostic criteria of ICD-11 [37]. A study has already demonstrated that men with anorexia nervosa pay less attention to their weight than women with the same disorder [38], although body dissatisfaction can result in maladaptive behaviours regardless of gender and lead to the manifestation of eating disorder symptoms in the long term [39]. A comprehensive social media analysis of posts by male content creators with a mesomorphic body type on the platforms Instagram and TikTok should provide a more nuanced understanding of conspicuous behaviours and lifestyle aspects of this target group. The prevailing diagnostic criteria for anorexia nervosa as delineated in ICD-11 were utilised as a foundational framework for the analysis, with the objective of discerning tendencies towards eating disorders within the population of male content creators. This also raises the question of the extent to which the existing criteria adequately reflect gender-specific characteristics.

## Materials and methods

### Literature research

The accompanying narrative literature research was conducted via Google Scholar, PubMed, and ResearchGate, limited to German and English sources. Relevant search terms in both languages were combined using Boolean operators (including eating disorder, anorexia nervosa, men, males, social media, social networks, body image, body perception, fitspiration, muscle dysmorphia, muscle addiction, Instagram, TikTok). Literature was managed using Citavi (version 6.18, Swiss Academic Software).

### Ethics

The dataset for this study was obtained from publicly available Instagram and TikTok accounts. After consultation with the Ethics Committee of the University of Applied Sciences Münster, no ethical approval was required. To ensure that the research was conducted in an ethical manner, all data was de-identified to protect the identities of the individuals and brands included.

### Social media analysis

The content analysis of a selection of social media platforms was conducted in a systematic and rule-based manner, encompassing the collection and evaluation of texts. Texts were interpreted to reveal both manifest and latent meanings, through the process of decomposition as defined by Berelson et al. [40]. The identification of the most popular social media platforms among young people was conducted through consultation of the JIM (youth, information, media) study series from 2022, which includes Instagram and TikTok [41]. For the purposes of clarity and scope, the data collection was limited to these platforms. Separate research accounts were set up to collect the data, using a trained algorithm. To this end, relevant hashtags such as #leanbody, #leanmuscle #leangain, #beforeandafterweightloss, #leanmalemodel, #nobodyfat, #sixpack, #bodybuilding, #fitness, #fitnessmodel and #muscle were used in the search function to generate corresponding posts on the discover page. The data was manually and systematically coded by one researcher. To ensure consistency and quality, a second researcher randomly cross-checked a subset of the coded material [42]. The analysis was based on a non-reactive, covert observation of publicly available social media posts to ensure that the content creators’ behaviour would be unaffected [43]. The process of data collection and analysis was divided into two central phases. The initial phase entailed the documentation of a four-week observation period of the published content of the sample. The second phase involved the documentation and analysis of existing profiles of content creators, as well as a review and adaptation of the diagnostic criteria for anorexia nervosa according to ICD-11.

### Sample selection

The objective of the sample selection process was to identify male content creators with mesomorphic, athletic body types, characterised by visible and defined musculature. These individuals correspond to the male ideal of beauty described in the extant literature [44, 45]. Content creators suitable for this analysis were selected exclusively on Instagram via the algorithm-driven discover page (see previous section) and through a supplementary search using a snowball sampling system based on the profiles that were identified. As the exact size of the population could not be determined, it was impossible to calculate the required sample size. Accordingly, a sample of between 25 and 30 individuals was chosen. Following the selection of individuals who fulfilled the primary criterion of muscular, athletic-lean body type, secondary inclusion criteria were then examined (see Table 1).

**Table 1:** Inclusion criteria for the content creator selection by category

Category	Inclusion criteria
Age	The selected candidate must be within the age range of 18 to 30 years.
Language of contribution	The contributions are primarily composed in English or German.
TikTok account	The selected individual is required to utilise a TikTok account that can be definitively attributed to them.
Number of followers	The selected individual must have a minimum of 25,000 followers on both Instagram and TikTok, thus being categorised as a power middle class or macro influencer.^1^
Account accessibility	The Instagram and TikTok profile is publicly accessible.
Account holder	The selected person is defined as an individual, rather than a group or company.

^1^ Micro-influencers: approx. 5,000 – < 25,000 followers; power mid-range influencers: approx. 25,000 – < 100,000 followers; macro-influencers: from approx. 100,000 followers [46]

Competitive and elite athletes, as well as bodybuilders at the competitive level were excluded, as they usually follow strict training and nutrition regimes that could distort the results. Profiles that showed illegal substance abuse and inactive profiles were also excluded.

### Data collection (Phase 1)

The data collection, spanning a period of four weeks, was conducted in the months of June and July of 2023. The collection of data encompassed newly published reels, stories, and posts on Instagram, while on TikTok, the focus was on stories and video posts. Existing OnlyFans accounts of content creators were also recorded, as the platform generally focuses on the content creator’s body [47] and links to eating disorders could be possible. The sexual orientation of the subjects was identified based on self-disclosed information available on their social media profiles. If no such information was found, the category was coded as ‘No information’. Also, the age of the content creators was extracted from self-reported information on their social media profiles (e.g. in bios, posts or highlights). Where this information was unavailable, publicly accessible online sources were used to determine the age as accurately as possible. Furthermore, all texts accompanying image posts, such as captions, were evaluated. Consequently, a unit of analysis was defined as a format-independent image post inclusive of the underlying caption. All published posts were systematically recorded and archived at 24-hour intervals. Following the collection of the raw data from the observation period, a plausibility check was conducted to ensure the data was complete and had been entered correctly. Content related to nutrition, exercise, or body presentation, whether spoken in the videos, shown as on-screen text, or included in the captions, was transcribed. Following this, categories were formed for the content recorded, using a combination of inductive and deductive operationalisation procedures based on the approach developed by Mayring (2022) [48]. The category systems are each based on coding guidelines that allowed for precise execution of categories, coding rules, descriptions and anchor examples, so that an accurate assignment and quantification of the content could be carried out [49]. Posts were considered eligible if they could be assigned to at least one main category of ‘nutrition’, ‘exercise’ and ‘body presentation’, with their respective subcategories (see Supplementary Table S1), developed based on the literature and the content of the observation period. In addition to these main coding categories, the above-mentioned descriptive characteristics (i. e. the presence of an Onlyfans account or sexual orientation) were also recorded. These variables were not part of the systematic coding scheme but were noted to allow for exploratory consideration of potential associations with the main research questions. Influences of childhood and adolescence were also not included as a separate coding category. However, the profile analysis retrospectively examined body shape development over the course of childhood and adolescence up to adulthood based on photographs and videos (see the following section). The coding guidelines were checked and optimised via pre-tests on two content creators to enable consistent coding [49]. In order to ensure reliability, the entire units of analysis from the observation period were recoded, and any coding errors identified were corrected [49]. Furthermore, a thematic qualitative examination was carried out to better understand and interpret specific behaviors of the content creators. Eligible activities and statements by the content creators were transcribed and categorized into the main categories of nutrition, exercise, and body representation using an inductively developed category system (see Supplementary Table S2). This approach complements the quantitative findings and provides deeper insights into the behaviors of the content creators.

### Development and testing of category systems to identify tendencies of eating disorders (Phase 2)

Two category systems were also developed as part of the profile analysis. The first system is utilised to quantify the fulfilment of the diagnostic criteria according to the ICD-11 diagnostic criteria for anorexia nervosa (coding 6B80). The second system was developed specifically to quantify the criteria adapted to the male sex based on the literature research (see section ‘Literature research’) (see Supplementary Tables S3 and S4). To ensure systematic and consistent coding, two coding guidelines were developed for use in the context of social media analysis, which were checked with the help of a pretest. During this pretest, the coding rules were reviewed using recorded content from two content creators, allowing adjustments to the coding descriptions and categories. Afterwards, the diagnostic criteria were reviewed as part of the profile analysis, which covered the content of the profiles up to the start of the research and also included the content recorded from the observation period. The analysis encompassed publications in various forms, including posts, reels, stories, story highlights, and captions, meticulously examining them against the predefined criteria. The results of this analysis were then systematically coded. The presence of tendencies towards anorexia nervosa is indicated by the fulfilment of all the main criteria by a content creator in the sample. Furthermore, the profile analysis was utilised to retrospectively examine body shape development over the course of childhood and adolescence, up to the point of adulthood, as determined by published posts. The context of this matter is that eating disorders commonly develop during early adolescence [50, 51]. To this end, photographs and videos from childhood and adolescence were analysed, with these media increasingly being found on online platforms. This comprehensive approach is expected to facilitate the identification of potential associations and patterns between early childhood and adolescent body shape and the subsequent development of eating disorder behaviour.

### Data evaluation

The data obtained from the profile analysis and the characteristics employed to describe the sample were transferred to the Statistical Package for Social Sciences (SPSS) version 29.0.1. The variables were then sorted, labelled and value labels were set. For the purpose of sample description, data pertaining to age, sexuality, origin, and so forth, were analysed using descriptive statistics. To identify potential patterns, correlations between different categories were established using cross-tabulation and evaluated using the Chi-square test [52]. The correlations between sexual orientation, the existence of an OnlyFans account, origin, body weight in childhood and body weight in adolescence, and the fulfilment of the diagnostic criteria according to ICD-11 and the fulfilment of adapted criteria for an eating disordered behaviour pattern were examined. The significance level was set at *p* ≤ 0.05. The ensuing results were presented based on the p-value and degree of freedom (df).

## Results

### Characterisation of the sample

28 male content creators were included in the sample. Two profiles (7.1%) were excluded during the observation period due to inactivity (< 2 posts per week). The key aspects of the sample characterisation are summarised in Table 2.

**Table 2:** Characteristics of content creators and their profiles

Characteristic	Value
Number of content creators	26
Age (in years)^1^	18–28 (21.7 ± 3.2)
Followers on Instagram	43.000–1.100.000 (287.880 ± 302.784)
Followers on TikTok	39.100–13.300.000 (1.603.865 ± 2.985.215)
Sexual orientation^2^	
Heterosexual	46.2%
Homosexual/bisexual	34.6%
No information	19.2%
Occupation	
Content creator	100.0%
Model	57.7%
Other	15.4%
Origin	
North America	53.8%
Europa	46.2%
Language	
English	96.2%
German	3.8%
OnlyFans account	
Available	46.2%
Not available	53.8%

^1^ Information according to self-disclosure in the social media or other online sources

^2^ Information according to self-disclosure in the social media

### Frequency, format and relevance of social media posts

A total of 2,985 posts were recorded during the observation period. The most prevalent format was identified as Instagram stories, accounting for 65.7% (n = 1,916) of the total. This was followed by TikTok videos, which constituted 15.0% (n = 449), and Instagram posts, accounting for 14.9% (n = 446). The least prevalent format was identified as Instagram reels, accounting for 4.3% of the total (n = 129). It is noteworthy that TikTok stories were not published. A total of 2,536 posts were published, constituting approximately 85% of all published content. For the evaluation, 2,035 posts (68.7% of the total posts analysed) were classified as eligible by at least one characteristic of the variables ‘nutrition’, ‘exercise’ or ‘body presentation’. The format with the most eligible posts was Instagram stories (59.8%, *n* = 1217), followed by TikTok videos (18.4%, *n* = 375), Instagram posts (16.4%, *n* = 334) and Instagram reels (5.4%, *n* = 109). The mean number of posts published during the observation period was 115 ± 76.1 (min = 26; max = 291) per content creator.

### Social media content on nutrition

A total of 11.3% (n = 335) of all posts included food, of which approximately half (49.2%, n = 165) was categorised as eating out. In 42.6% (n = 185) of the posts, the food was unprocessed or minimally processed, while the remainder was categorised as processed or heavily processed food. The food categories most frequently documented were ‘fruit and vegetables’ (15.8%, n = 95), ‘water/tea/unsweetened drinks/coffee’ (15.8%, *n* = 95) and ‘alcoholic drinks’ (15.5%, *n* = 93). The presence of food supplements was identified in 2.1% (*n* = 13) of food-related posts. These included various beverages (e.g. protein shakes, hydration and BCAA drinks) as well as protein powders, pre-workouts, protein bars and protein cornflakes. Furthermore, the following nutritional recommendations were documented:

- Recommendation of an adequate water supply (*n* = 2)
- Recommendation of a high-protein diet (*n* = 3)
- Discipline regarding nutritional behaviour (*n* = 1)
- Consumption of unprocessed foods with a high nutrient density (*n* = 3)
- Use of zero and light products (*n* = 2)

In addition, the terms ‘ketogenic diet’ (n = 1) and ‘cutting phase’ (*n* = 1) were identified as explicit mentions in connection with diets.

### Social media content on physical activity

**Fig. 1:**
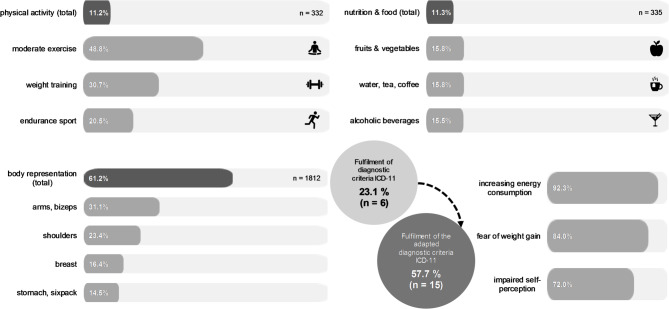
Content shares of the categories sport, nutrition and body presentation and ICD-11 fulfilment levels inclusive the adapted diagnostic criteria

In 11.2% (*n* = 332) of the posts, indications of physical activity were identified. Of these, 20.5% (*n* = 42) were classified as endurance sports, such as jogging, hiking or tennis. In 30.7% (*n* = 63) of the posts, weight training was performed. The most prevalent categorisation (48.8%, *n* = 100) was light physical activity, such as yoga and walking (see Fig. 1). The motivations for engaging in physical activity included the pursuit of a desired body shape and muscle strength, sport as a coping strategy, health benefits and sport as a compensatory measure for energy surplus and unhealthy food consumption. Recommendations for exercise were also issued to followers. The dissemination of training plans for different muscle groups, recommendations for endurance training and suggestions for integrating everyday movement were identified. In this context, the importance of daily exercise, improving performance and the associated discipline were emphasised.

### Social media content for presenting the body

61.2% (n = 1,812) of the posts exhibited a deliberate staging of the body. This category was dominated by posts taken by other people or using a self-timer (63.4%, n = 1,148). 22% (n = 400) of the posts were selfies, while 14.6% (n = 264) of the posts were classified as mirror image self-portraits. Furthermore, 59.9% (n = 1,774) of the posts showed unclothed body parts. The body parts most frequently depicted were unclothed arms (31.1%, n = 1,571), unclothed shoulders (23.4%, n = 1,179) and the unclothed breast (16.4%, n = 827). The categories of unclothed stomach (14.5%, n = 731), legs (10.8%, n = 547), back (3.7%, n = 184) and buttocks (0.2%, n = 9) were less frequently recorded (see Fig. 1). The category ‘visible musculature’ was also fulfilled in 55.7% (*n* = 1,649) of the posts.

### Profile analysis

According to the profile analysis, 23.1% (n = 6) of the content creators would fulfil all diagnostic criteria for anorexia nervosa according to ICD-11. Conversely, the adapted criteria for males would be met by 57.7% (n = 15) of the content creators. This indicates that a further nine content creators with a tendency towards anorexia nervosa could be identified using the modified criteria for anorexia nervosa. The most prevalent symptoms according to the ICD-11 criteria were ‘increased energy expenditure’ (92.3%, n = 24), ‘fear of gaining weight’ (84.0%, n = 21) and ‘disturbed self-perception of body weight and body shape’ (72.0%, *n* = 18). In contrast, all content creators met the criterion ‘high lean body mass and low fat mass’ for the modified criteria, followed by the criteria ‘increase in energy expenditure’ (92.3%, *n* = 24) and ‘fear of an increase in fat mass’ (88.5%, n = 23).

A statistically significant association was not identified between sexual orientation and the fulfilment of the diagnostic criteria for anorexia nervosa according to ICD-11 (*p* = 0.676, df = 1). A similar outcome was observed in the relationship between fulfilment of the adapted diagnostic criteria and the sexual orientation of the participants (*p* = 0.899, df = 1). Furthermore, the investigation revealed no statistically significant association between the possession of an OnlyFans account and a diagnosis of anorexia nervosa according to the ICD-11 criteria (*p* = 0.473, df = 1) or when the adapted criteria were applied (*p* = 0.951, df = 1). Additionally, no statistically significant correlation was identified between body weight during childhood and the tendency towards anorexia nervosa according to ICD-11 (*p* = 0.643, df = 2) or the adapted criteria (*p* = 0.422, df = 2). While no statistically significant association between body weight in adolescence and the trends of anorexia nervosa was found according to ICD-11 (*p* = 0.222, df = 2), a significant association could be demonstrated according to the adapted criteria (*p* = 0.047, df = 2). A detailed analysis by weight range revealed a significant correlation with eating disorders in both the potentially lower normal weight range (*p* = 0.026, df = 1) and the potentially normal weight range (*p* = 0.013, df = 1).

## Discussion

As demonstrated in the extant literature, the staging of the body (61.2%) was predominantly characterised by the utilisation of revealing clothing and figure-hugging shots, a phenomenon that has been widely documented. This tendency aligns with the notion that permissiveness is concomitant with recognition and a substantial social media reach [9]. Beyond the use of images depicting revealing clothing, a notable emphasis was placed on the portrayal of the mesomorphic body ideal in male content creators, with over half (55.7%) of the published content featuring overt muscular displays. This tendency is accompanied by the frequent documentation of physical changes associated with body-checking behaviours. This has been particularly documented in individuals with anorexia nervosa in the context of a body schema disorder [53–55]. It also occurs in the context of fitspiration content [56] and indicates an overvaluation of body shape and weight [57]. Furthermore, the content creators’ observational behaviour, including body-related statements and associated self-doubt, demonstrates tendencies of muscle dysmorphia, characterised by a persistent fear of an insufficiently muscular body [27, 30]. Despite already conforming to the mesomorphic body ideal, these individuals frequently engage in self-control and self-denial to approach the desired body type [58]. These observations align with the findings of Badenes-Ribera et al. (2019), who identified a positive correlation between muscle dysmorphia and eating disorder symptomatology [59].

In addition to recommendations regarding dietary intake and presentations on the subject, nutritional advice was provided to followers. This included the consumption of low-calorie foods, primarily in their unprocessed form, and the utilisation of light products wherever possible. This restriction of energy intake is indicative of the pursuit of a low body fat percentage. Furthermore, the integration of the cutting phase was emphasised by one content creator. The aforementioned approach is described in the literature, particularly in the context of muscle dysmorphia [60]. The deliberate adoption of specific eating behaviours in the context of muscle building is recognised as ‘muscularity-oriented disordered eating’ [61] and occurs particularly in combination with muscle-building training [30]. The consumption of alcoholic beverages (16%), heavily processed plant-based products (12%), and sweet beverages (7%) is contradictory in this context. These food groups, which are typically high in calories, are often avoided due to the high calorie content in the presence of an eating disorder [62]. Another contradictory phenomenon observed in the documented eating behaviour, as compared to extant literature, pertains to the elevated prevalence of out-of-home consumption exhibited by content creators afflicted with muscular dysmorphia [61]. In the relevant literature, both in the context of classic anorexia nervosa [63] and in the context of muscle dysmorphia, an avoidant attitude towards meals in social contexts is described [61]. It has also been observed that those affected avoid eating outside the home due to a lack of knowledge about calorie content [61]. This deviant behaviour could be related to their professional activity as content creators, which may demand public appearances or food-related content. Moreover, it is noteworthy that content creators who offer product or nutrition recommendations exert a significant influence on the purchasing decisions of young target groups, particularly due to their perceived credibility [64].

In addition to nutrition-related behaviours, content creators identified a high level of sporting activity as a means of maintaining the existing body type. Weight training was frequently linked to enhanced performance, including increased muscle strength and mass. Moreover, exercise was observed as a compensatory measure in the event of an energy surplus or following the consumption of calorie-dense foods, a behaviour that has also been observed in individuals diagnosed with anorexia nervosa [65]. Furthermore, the increase in everyday exercise was explicitly passed on to followers as a recommendation. While these social media contentions are ostensibly designed to motivate consumers to adopt a healthy lifestyle, they frequently result in the internalisation of a thin and athletic body ideal, followed by disturbed eating behaviour and compulsive exercise [66, 67]. Excessive exercise is frequently interpreted in a positive manner, particularly in male sufferers, and consequently, it is often underestimated and trivialised as a symptom of eating disorders [68].

The patterns presented thus far suggest a partial correlation with eating disorder-related behaviours. As social media usage increases, both conscious and unconscious exposure to such content may also increase [1]. Children and adolescents in particular have been observed to internalise the behaviours presented by content creators, integrating them into their own everyday lives [69]. This influence can lead to the adoption of certain practices in the areas of health, fitness or body image being uncritically adopted and manifesting in maladaptive behaviour, including the onset of an eating disorder [70]. A meta-analysis by Holland and Tiggemann (2016) found a link between social media use and eating disorder behaviour [22]. Wilksch et al. (2020) also observed that the more time spent on Instagram, the higher the susceptibility to disordered eating behaviour, especially among young girls [71].

As described, revealing clothing and body-focused content is an essential part of the social media accounts examined. Since Instagram and TikTok prohibit nudity in their community guidelines [72, 73], many content creators switch to platforms like OnlyFans to publish or sell erotic to pornographic content [47]. OnlyFans accounts were found for 46.2% of the content creators examined. The concept of OnlyFans suggests that the body and its presentation are staged even more intensely than on Instagram and TikTok and for monetary purposes, further increasing the pressure on recipients to maintain and optimise their bodies.

The present analysis, however, was unable to confirm the assumption that there is a correlation between sexual orientation and tendencies towards eating disorders. In homosexual and bisexual individuals, a high ideal of appearance and physical presentation has been described, which has been hypothesised to trigger increased symptoms of eating disorders within the group [74]. However, this could not be confirmed in the present sample, as the group of content creators, regardless of sexual orientation, appears to be subject to comparable beauty ideals. The results of Gueguen et al. (2012) suggest that men with eating disorders often had a premorbid history of overweight in childhood and adolescence [75], while the findings from the social media analysis suggest that both low and normal body weight in adolescence could be associated with an increased risk of eating disordered behaviour.

The role of body weight is also critically examined within the ICD-11 criteria, particularly in relation to the mesomorphic body ideal. In this context, the diagnostic requirement of a significantly reduced body weight according to ICD-11 is to be regarded as inappropriate in relation to a mesomorphic body ideal, since men, due to their increased muscle mass, can be in the BMI (body-mass-index) range of normal weight (18.5–24.9 kg/m^2^). In the present analysis, 68% of the content creators were classified as having a normal weight based on a visual assessment, although some had a very low body fat percentage or a slim, muscular build. Consequently, the criterion was modified to a ‘high lean body mass and low fat mass (< 10%)’. The ICD-11 diagnostic criterion of rapid weight loss was only demonstrated in 24% of cases. As Strober et al. (2006) previously described, body weight is not the primary focus for male sufferers [38]. Accordingly, the majority did not show weight loss, but only a change in body composition with no weight loss or with weight gain in the form of muscle mass. The above aspects led to the development of the modified criterion ‘Rapid change in body composition within 3 to 6 months (high lean body mass, low fat mass)’. Another diagnostic criterion according to ICD-11 is an existing disturbed self-perception of those affected with regard to body weight or body shape. Despite their low body weight, subjects frequently perceived it to be either normal or excessive. Due to the limitations of the social media analysis, which does not permit direct questioning of the content creators, specific behaviours were recorded in order to assess the criterion. In this context, dissatisfaction and derogatory comments about one’s own body shape or weight were documented, as were excessive measures of self-optimisation, including excessive physical activity or dieting. The criterion of a disturbed self-perception was also integrated into the adapted survey criteria. However, the focus was not on low body weight, but on the pursuit of the mesomorphic body ideal. The results of the study indicate that the gender-specific adapted criteria led to the identification of nine additional content creators who exhibited tendencies towards eating disorders, such as anorexia nervosa, which were not detected by the standard diagnostic criteria according to ICD-11. Conversely, those already identified by the ICD-11 diagnostic criteria were also identified by the adapted criteria. This finding suggests that the adapted criteria do not neglect those affected, but rather enable the additional identification of male individuals with a tendency towards eating disordered behaviour. The assessment was based on snapshots and targeted insights provided by the sample. Despite these limitations, gender-specific behaviours in men could be identified that indicate tendencies towards restrictive eating and behavioural patterns.

Further limitation of the analysis is the limited sample size, which is due to the high effort required for the visual and textual evaluation. Moreover, the sample is not representative of the total population, since it was obtained through a feature-specific selection process and the population of male content creators cannot be determined. These aspects result in limitations regarding the generalisability of the results. Furthermore, the sample characterisation was based on publicly available information, and since there was no direct contact with the sample, the information could not be validated by the content creators. The study’s scope was further constrained to the platforms Instagram and TikTok, excluding other social media sites such as YouTube. Additionally, the observation period was limited, which hinders the capture of seasonal and temporal shifts in behaviour. It should also be noted that social media is used as a means of targeted self-presentation by users and that only targeted snapshots are published. Accordingly, the method of social media analysis only allows a limited and selected insight into the actual reality of the content creators. Accordingly, the evaluation of the data was only possible on the basis of visual criteria. This approach impacts the quality of the data, but it also prevents distortions that may arise from selective observation. The generation of coding guidelines ensured reproducibility. Despite the limitations of the study, the methodological approach, combining qualitative and quantitative content analysis, provides a comprehensive insight into the target group’s eating, exercise and body image behaviours.

## Conclusions

The combination of a qualitative and quantitative content analysis in social media provides a comprehensive insight into the eating and exercise behaviour and body presentation of male content creators with an existing mesomorphic body ideal. The qualitative research approach allowed additional information to be generated, which in turn allowed in-depth insights. Furthermore, tendencies of eating disordered behaviour patterns could be illustrated by means of the profile analysis. Based on the present results, early education of male children and adolescents should take place in order to avoid critical behaviour patterns and utopian beauty ideals.

## Supplementary Information

Below is the link to the electronic supplementary material.

Supplementary Material 1

## Data Availability

The raw data (German-language) supporting the conclusions of this article will be made available by the authors on request.
