# Supplementary material for: Tendencies of eating disordered behaviours in male content creators: a social media analysis
Source: J Eat Disord. 2025 Sep 9;13:201. doi: 10.1186/s40337-025-01395-8 (PMC12418650; doi:10.1186/s40337-025-01395-8)
Supplement: Supplementary file 1 — Supplementary Material 1 [file 40337_2025_1395_MOESM1_ESM.docx]

***Supplementary***

***Table S1*** *Brief description of the coding guide (phase 1) for the quantitative analysis of posts on Instagram and TikTok during the four-week observation period*

| Category | Description |
| --- | --- |
| 1. *Formal categories* |  |
| Date | Recording the time of data collection |
| Format | Classification by format: Instagram post, story or reel, TikTok video |
| 1. *Nutrition* |  |
| Illustration of food | Entries showing one or more foods |
| Unprocessed foods, less processed foods | Categorisation into unprocessed/lightly processed vs. processed/heavily processed foods, based on the NOVA classification |
| Fruit/vegetables | Contributions with recognisable types of fruit or vegetables |
| Water/tea/unsweetened drinks/coffee | Contributions with unsweetened drinks |
| Animal products (unprocessed) | Contributions with little or unprocessed animal products |
| Vegetable products (unprocessed, minimally processed) | Contributions with little or unprocessed plant-based products |
| Oils/greases | Contributions with oils or fats |
| (Heavily) processed foods | Categorisation into unprocessed/lightly processed vs. processed/heavily processed foods, based on the NOVA classification |
| Alcoholic beverages | Contributions with alcoholic beverages |
| Sweet drinks | Contributions with sweetened beverages |
| Sweets | Posts with confectionery |
| Animal products (processed) | Contributions with processed or highly processed animal products |
| Vegetable products (processed) | Contributions with processed or highly processed plant products |
| Fast food/ convenience products/ snacks | Contributions with fast food, ready-made products or snacks, including quickly prepared or packaged foods |
| Sauces/dips | Contributions with sauces or dips |
| Food supplements | Contributions with supplements or fortified foods |
| Out-of-home consumption | Contributions with food consumption outside the home |
| *Movement* |  |
| Presentation/telling of physical activity | Contributions with physical activity or story about it. Subdivision into endurance sports, weight training, light activity |
| Endurance sport | Contributions with physical activity, allocation to endurance sports |
| Weight training | Contributions with physical activity, allocation to strength sports |
| Light physical activity | Contributions with light physical activity, neither clearly categorised as strength nor endurance sports |
| Person in a sporting environment without sporting activity | Contributions of the person in the sporting environment without visible activity |
| *Body representation* |  |
| Staging the body | Body images in figure-hugging clothing or undressed. Subdivision into mirror selfie, selfie and self-timer or self-timer shot |
| Display of unclothed body parts | Illustration of unclothed body parts. Subdivision into arm/ shoulder/ chest/ abdomen/ buttocks/ leg |
| Visible musculature | Contributions with clearly recognisable musculature |
| Statements regarding body changes | Statements about body changes |

***Table S2*** *Brief description of the coding guide (phase 1) for the qualitative analysis of the observation period of posts on Instagram and TikTok within the four-week observation period*

| Category | Description of the |
| --- | --- |
| *Nutrition* |  |
| Type of food supplements | Presentation of food supplements, supplements and fortified foods |
| Dietary recommendations | Statements on dietary recommendations and advice, conspicuous behaviour that encourages imitation and repeated conspicuous dietary behaviour. |
| Dietary recommendations | Recommendations or implementation of weight-reduced diets |
| *Movement* |  |
| Motivation for physical activity | Recording statements about motivational reasons for physical activity |
| Exercise recommendations | Suggestions for physical exercises or increasing physical activity for followers |
| *Body representation* |  |
| Statements regarding body changes | Information on body changes already made or planned |
| Statements on self-worth | Positive or negative statements about your own self-worth |

***Table S3*** *Brief description of the coding guide (phase 2) for the quantitative evaluation of the profiles based on the diagnostic criteria according to ICD-11*

| Category | Description of the |
| --- | --- |
| 1. *Significantly low weight* | BMI <18.5 kg/m² and/or noticeable weight loss over a period of 6 |
| Estimation of the body-mass-index (BMI) to <18.5 kg/m^2^ | Recording of BMI <18.5 kg/m² |
| Rapid weight loss | Visible decrease in body fat mass and definition of the musculature within 6 months. Written, verbal statements and visual images of the body can be used for the assessment |
| 1. *Detection of behaviours that prevent the return to normal weight* | Detection of behaviours that prevent the return to normal weight, such as reducing energy intake, purging behaviour or increasing energy consumption |
| Reducing the energy supply | Indications of a reduced calorie intake |
| Purging behaviour | Indications of purging behaviour |
| Increase in energy consumption | Indications of a targeted increase in energy consumption |
| 1. *Fear of gaining weight* | Statements about fears of gaining weight or behavioural patterns in this regard |
| 1. *Impaired self-assessment of body weight and body shape* | Information regarding impaired self-assessment of body weight and body shape |

***Table S4*** *Brief description of the coding guide (phase 2) for the quantitative evaluation of the profiles using customised target group-specific diagnostic criteria*

| Category | Description |
| --- | --- |
| 1. *Body composition* | Measurement of body composition or significant change in body composition |
| High lean body mass and low fat mass | Indications of a high lean body mass (LBM) and low body fat mass |
| Rapid change in body composition | Indications of a rapid change in body composition within 3 to 6 months |
| 1. *Behaviour to maintain a mesomorphic body shape* | Detection of behaviours that maintain the mesomorphic body shape |
| Reducing the energy supply | Indications of reduced calorie intake and/or a diet high in protein and/or regular use of dietary supplements to build muscle |
| Cleaning behaviour | Notes on cleaning behaviour |
| Increase in energy consumption | Excessive physical activity to maintain the mesomorphic body ideal |
| 1. *Fear of a change in body shape* | Fears of increasing fat mass and changes in body shape |
| 1. *Impaired self-assessment of body weight and body shape* | Indications of impaired self-assessment of body weight and body shape |
